# Description of a new *Hiroshia* species (Lepidoptera, Thyatiridae) from Hubei Province, China

**DOI:** 10.3897/zookeys.996.55002

**Published:** 2020-11-24

**Authors:** Hong Zheng, Gábor Ronkay, László Ronkay, Hui-Lin Han

**Affiliations:** 1 School of Forestry, Northeast Forestry University, Harbin, CH-150040, China; 2 Heterocera Ltd, H-11437 Budapest, Szt. István krt 4, Hungary; 3 Key Laboratory of Sustainable Forest Ecosystem Management-Ministry of Education, Northeast Forestry University, Harbin, 150040, China

**Keywords:** False owlet moth, identification key, male genitalia, morphology, new species, taxonomy

## Abstract

A third species of *Hiroshia* László, Ronkay & Ronkay, 2001, *H. shennongjiaensis* Ronkay, Ronkay & Han, **sp. nov.** is described from Hubei Province in China. The adult and the male genitalia of the new species are illustrated and compared with those of its congeners, *H. albinigra* László, Ronkay & Ronkay, 2001 and *H. nanlingana* Zhuang, Owada & Wang, 2014; an identification key based on the male genitalia is presented.

## Introduction

The genus *Hiroshia* László, Ronkay & Ronkay, 2001 was established for a single species, *H. albinigra* László, Ronkay & Ronkay, 2001 which occurs in the Mt. Fansipan, N. Vietnam. Subsequently, an allopatric sister-species, *H. nanlingana* Zhuang, Owada & Wang, 2014 was discovered and described from the Mt. Nanling in China (László et al. 2001, 2007; Zhuang et al. 2014). In this present study, a third species of the genus, *H. shennongjiaensis* Ronkay, Ronkay & Han, sp. nov. is described from Hubei Province, China, from an untouched forest area composed of broad-leaved forest, mixed coniferous woodland and shrubby undergrowth.

## Materials and methods

The examined material originates from the collections of the Institute of Zoology, Chinese Academy of Sciences. Dissection of the abdomen and genitalia follows Kononenko and Han (2007). The moths were photographed using the camera Nikon D700; the genitalia slides were photographed with an Olympus photomicroscope with Helicon Focus software, further processed in Adobe Photoshop CS6. The type-specimens of the new species are deposited in the collection of the Institute of Zoology, Chinese Academy of Sciences (**IZCAS**).

## Taxonomic account

### Genus Hiroshia

Taxon classificationAnimaliaDipteraTabanidae

László, Ronkay & Ronkay, 2001

BA38FC88-ECCC-59C3-8F30-DF9684F1B8E9

Hiroshia László, Ronkay & Ronkay, 2001, Acta Zoologica Academiae Scientiarum Hungaricae 47(1): 27–85. Type species. Hiroshia albinigra László, Ronkay & Ronkay, 2001 (type-locality: Vietnam, Fan-si-pan Mts. [MWM]).

### Hiroshia shennongjiaensis

Taxon classificationAnimaliaDipteraTabanidae

Ronkay, Ronkay & Han sp. nov.

2B10FAD9-FDDE-5D09-844D-0929D3142DE4

http://zoobank.org/24982942-1CEF-4D70-985A-AF638153D721

**Figures 1–4.**
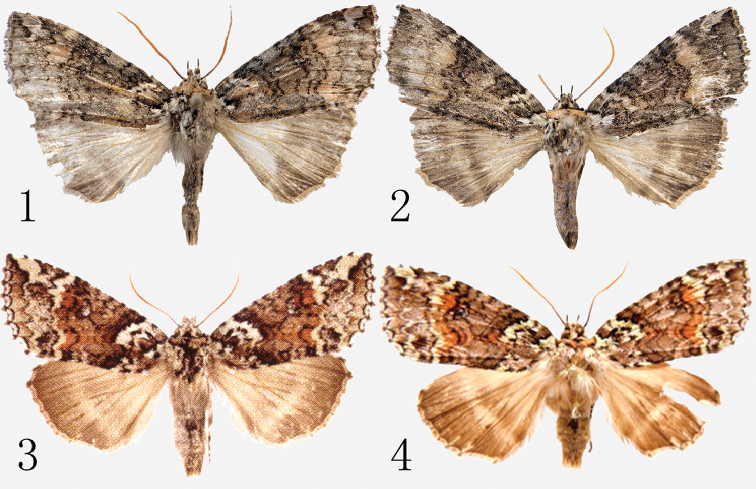
*Hiroshia* spp., adults **1***H. shennongjiaensis* sp. nov., male, holotype **2** ditto, male, paratype **3***H. albinigra*, male (László et al. 2007) **4***H. nanlingana*, male (after Zhuang et al. 2014).

Figures 1–2 , 6 , 8–10

#### Material examined.

***Holotype*.** ♂, China, Hubei Province, Badong County, Yanduhe town, Xiaoshennongjia village, altitude 1320 m, 26. iv. 2016, leg. J Yao & KD Zhao; gen. prep. No. hhl-4220-1; coll. IZCAS. ***Paratypes*.** 2 ♂, from the same site as the holotype, 28. iv. 2016, leg. J Yao & KD Zhao; coll. IZCAS.

#### Diagnosis.

The new species is very similar externally to *H. albinigra* (Fig. 3) and *H. nanlingana* (Fig. 4) by its size (wingspan 46–46.5 mm, those of *H. albinigra* and *H. nanlingana* are 46–51 mm and 48–51 mm, respectively), wing shape and main elements of the forewing pattern. The forewing is more unicolorous, without prominent whitish markings which are typical of the other two species of the genus. The distinguishing features are as follows: forewing ground color of *H. shennongjiaensis* rather monotonous graphite-grey, without prominent reddish or red-brown irroration (in *H. albinigra* and *H. nanlingana* with conspicuous red or reddish-brown suffusion in median area); apical patch darker, pale bluish-grey (in *H. albinigra* and *H. nanlingana* white or grey-white); submarginal area pale ochreous-brown to greyish-brown between postmedial and praeterminal lines (in *H. albinigra* and *H. nanlingana* prominently white and grey-white); orbicular stigma more visible, whitish with distinct blackish outline (in *H. albinigra* and *H. nanlingana* indistinct); and the hindwing basal area is paler, rather greyish-white (in *H. albinigra* and *H. nanlingana*, it is darker, stronger, suffused by light brown).

**Figures 5–7.**
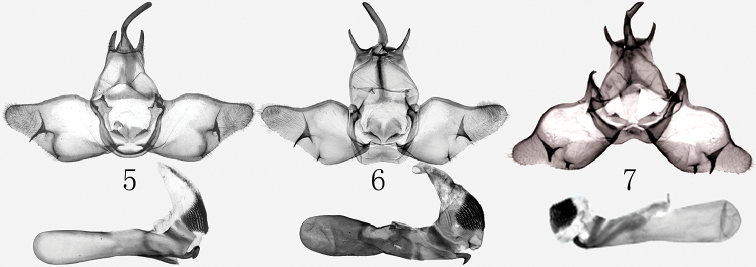
*Hiroshia* spp., male genitalia **5***H. albinigra*, male (László et al. 2007) **6***H. shennongjiaensis* sp. nov., male, holotype, gen. prep. No. hhl-4220-1 **7***H. nanlingana*, male (after Zhuang et al. 2014).

Configuration of the male genitalia of *H. shennongjiaensis* (Fig. 6) is more similar to that of *H. albinigra* (Fig. 5) than to *H. nanlingana* (Fig. 7) as both species have a rudimentary subbasal costal process which is very large and acutely pointed in *H. nanlingana*. The new species can be distinguished from its congeners by its thinner and longer socii (those of *H. albinigra* and *H. nanlingana* are shorter and thicker); broader and rather quadrangular tegumen (it is dorsally tapering and more or less trapezoidal in the other two species); thinner and stronger sclerotised fultura superior (it is broader and less strong in *H. albinigra* and *H. nanlingana*); larger dorsal sclerotised plates of the juxta and the acutely pointed and hook-like carinal tooth of the aedeagus (it is upturned and apically more or less rounded in the other two species). In addition, the basal process of the costa is smoothly arched in *H. shennongjiaensis* while it is shortly peaked in *H. albinigra*, and huge, thorn-like and apically hooked in *H. nanlingana*.

#### Description.

Adult (Figs 1–2). Male. Wingspan 46.0–46.5 mm. Pubescence of head mixed grey and light brown; labial palpi covered by grey scales at 1^st^ and 2^nd^ segments, 3^rd^ segment thin, finely scaled; antennae beige. Patagium beige; thorax covered by white and smoky black hair-scales. Abdomen dark grey, mixed with smoky black, greyish-brown and light brown scales. Ground color of forewing light graphite-grey, irrorated sparsely with smoky black and greyish-white scales; basal dash white or whitish-grey marked by blackish line; crosslines double and waved; basal line black, its inner line distinct and excurved, outer line thin and arched; antemedial line double, black, parallel and approaching, filled with white and pale grey scales; median fascia narrow, dark grey sinuous; postmedial line double, blackish-grey, incurved at cell and at veins CuA2-3A, its inner line close to outer line of median line; area between postmedial and preterminal lines pale ochreous-brown to greyish-brown; preterminal line black and discrete, arched; subterminal line finer, crenellate, weakly arched, incurved, distinct and rather broad at apex area, with light bluish-grey suffusion along its inner side; light patch of termen irregularly cuneiform, light bluish-grey; terminal line black, finely laced, incurved between veins; fringes greyish-brown and mixed darker brown; basal line area white; orbicular stigma small, round, whitish, with black frame; reniform stigma flat-cashew shaped. Hindwing basal area greyish-white, outer part of wing stronger greyish-brown to smoky grey suffused; transverse line broad and diffuse, slight incurved at CuA2-3A; discal spot obsolete; marginal area wide, dark smoky grey; fringes brown. Female unknown.

***Male genitalia*.** (Fig. 6) Uncus finger-shaped and sclerotized, weakly smooth curved at basal part; socii separated from uncus, straight, slender cone shaped, ca 5/9 as long as uncus, apically finely pointed. Tegumen broad, quadrangular, membranous, with slender and thick ventral edge; vinculum rather shortly U-shaped, moderately sclerotized, sunken at bottom. Juxta formal hat-shaped, sclerotized, sunken at dorsal margin. Fultura superior narrow and prominently sclerotized, reversed T-shaped. Valva irregular quadrangular; costal margin slender and thick, with a bulge ca 1/5 times as long as valva, then smoothly incurved, the process of basal costa smoothly arched; sacculus broad, swollen, shorter than half of valva, process of sacculus apically tiled; cucullus blunt round, densely covered long hair; harpe asymmetrical, strongly sclerotized, flat and triangular shaped, left one slightly arched, long, extend out of ventral margin, right one shorter than left one; clasper strongly sclerotized, very short spike-shaped, and extending towards saccular margin. Aedeagus long and tubular, straight, coecum swollen, ca ½ times as long as aedeagus; carina strongly sclerotized, trapezoid ring-shaped, dorso-lateral process hook-shaped and apically acute, the subprocess plate arched; vesica broader, with short and broad basal dorsal diverticulum, a large cornuti field consisting of separate, short, acute spinules, and a narrow band of minute spiculi extending towards ductus ejaculatorius.

***Female genitalia.*** Unknown.

#### Distribution.

**Figures 8–10.**
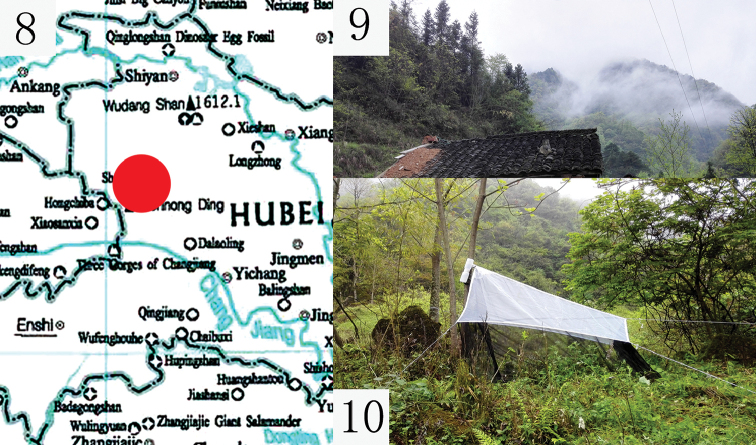
**8** Map showing collection site of *H. shennongjiaensis* sp. nov. **9–10** Both sides of the collection site composed of mainly broad-leaved forest, mixed conifers and shrubberies.

China (Hubei: Badong) (Fig. 8).

#### Etymology.

The species name “*shennongjiaensis*” refers to the type-locality in the Shennongjia National Nature Reserve in Hubei Province.

#### Bionomics.

The new species inhabits broad-leaved forest, mixed with conifers and shrubberies, at ca 1300 m altitude in the southern part of the Shennongjia National Nature Reserve (Figs 9, 10). This area is located in the western part of Hubei Province and is close to the Dabashan National Nature Reserve. The three known specimens were collected in April.

### Key to the species of the genus *Hiroshia* László, Ronkay & Ronkay, 2001 based on the male genitalia

**Table:** 

1	Socii close to uncus; basal costal process with smaller or larger apical thorn; carinal process apically rounded	**2**
–	Socii separated from uncus; basal costal process small, apically rounded; carinal process acutely hooking	*** shennongjiaensis ***
2	Basal costal process with short apical peak, vinculum evenly rounded	*** albinigra ***
–	Basal costal process with huge, apically curved thorn; vinculum medially deeply incised	*** nanlingana ***

## Supplementary Material

XML Treatment for Hiroshia

XML Treatment for Hiroshia shennongjiaensis
